# Vulnerability of the Oceanic Whitetip Shark to Pelagic Longline Fisheries

**DOI:** 10.1371/journal.pone.0141396

**Published:** 2015-10-22

**Authors:** Mariana Travassos Tolotti, Pascal Bach, Fábio Hazin, Paulo Travassos, Laurent Dagorn

**Affiliations:** 1 Institut de Recherche pour le Développement, UMR MARBEC (IRD, Ifremer, Univ. Montpellier, CNRS), Avenue Jean Monnet CS 30171, Sète cedex, France; 2 Programa de Pós-graduação em Oceanografia, Universidade Federal de Pernambuco, Departamento de Oceanografia, Recife—PE, Brasil; 3 Departamento de Pesca e Aquicultura, Universidade Federal Rural de Pernambuco, Recife—PE, Brasil; UC Santa Cruz Department of Ecology and Evolutionary Biology, UNITED STATES

## Abstract

A combination of fisheries dependent and independent data was used to assess the vulnerability of the oceanic whitetip shark to pelagic longline fisheries. The Brazilian tuna longline fleet, operating in the equatorial and southwestern Atlantic, is used as a case study. Fisheries dependent data include information from logbooks (from 1999 to 2011) and on-board observers (2004 to 2010), totaling 65,277 pelagic longline sets. Fisheries independent data were obtained from 8 oceanic whitetip sharks tagged with pop-up satellite archival tags in the area where longline fleet operated. Deployment periods varied from 60 to 178 days between 2010 and 2012. Tagging and pop-up sites were relatively close to each other, although individuals tended to travel long distances before returning to the tagging area. Some degree of site fidelity was observed. High utilization hotspots of tagged sharks fell inside the area under strongest fishing pressure. Despite the small sample size, a positive correlation between tag recorded information and catch data was detected. All sharks exhibited a strong preference for the warm and shallow waters of the mixed layer, spending on average more than 70% of the time above the thermocline and 95% above 120 m. Results indicate that the removal of shallow hooks on longline gear might be an efficient mitigation measure to reduce the bycatch of this pelagic shark species. The work also highlights the potential of tagging experiments to provide essential information for the development of spatio-temporal management measures.

## Introduction

The oceanic whitetip shark (*Carcharhinus longimanus*, OCS) is a tropical predator [1,2] commonly taken as bycatch in pelagic fisheries using longlines, gillnets, and purse seines [3]. Inadequate monitoring hampers the assessment of stock status for this species, but there is a broad consensus that populations are decreasing [4,5]. Concerns regarding the conservation of oceanic whitetips have risen substantially during the past decade due to increasing fishing pressure throughout the species range and inadequate catch monitoring, associated with an acute lack of knowledge on the behavior and ecology of the species. In response to an evident, and in some cases drastic, decline of abundance trends, Tuna Regional Fisheries Management Organizations (RFMOs), responsible for the management of tuna fisheries and associated species, adopted a series of strong measures to bolster conservation efforts for the oceanic whitetip shark (International Commission for the Conservation of Atlantic Tuna Rec. 10–07; Inter-American Tropical Tuna Commission Rec. C-11-10; Western and Central Pacific Fisheries Commission CMM 11–04; Indian Ocean Tuna Commission Res. 13–06). These measures essentially banned the take of oceanic whitetip sharks by prohibiting the retention, landing or storing of the whole carcass or any part thereof. The oceanic whitetip is the only pelagic shark species covered by such measures across all oceans. The species was also recently included in CITES appendix II (March 2013, CoP16 Prop. 42).

Under current fishing practices, however, the incidental capture of this species is difficult to be avoided. Furthermore, many individuals are dead or dying by the time the fishing gear is retrieved. As such, the measures currently in place do little to alleviate fishing mortality, which continues to impact oceanic whitetip populations across all oceans. The development of mitigation measures capable of not only reducing their catch rates but also increasing their post-release survival are therefore crucial for improving population levels [6]. In ecological terms, the vulnerability of a species depends upon two factors: the sensitivity of the species to an external factor and how extensive the exposure to this hazard is [7,8]. Shark species are known to have low rebound capacities resulting directly from their life history traits of slow growth, late maturation and low fecundity. These biological characteristics make this group much more sensitive to overexploitation than teleost fishes [9–11].

The present study assesses the vulnerability of the oceanic whitetip shark by analyzing the extent of its fishing exposure (accessibility) to pelagic longline fisheries. The Brazilian tuna longline fleet, operating in the equatorial and southwestern Atlantic, is used as a case study. The overall goal is to provide information needed to aid in the design of management measures and improve conservation efforts for this species. Both fisheries dependent (fisheries logbooks, observers) and independent (electronic tags) datasets were used as complementary sources of information. This integrating approach provided a better understanding of the interactions between oceanic sharks and this fishery. Vulnerability was assessed with regard to both spatial dynamics (horizontal) and depth distribution (vertical) of both sharks and longline gear, taking into consideration seasonality and strategies in the fishery.

## Material and methods

### Ethics Statement

The capture, handling and tagging of oceanic whitetip sharks were conducted during commercial fishing operations and did not require specific ethical approval according to national regulations. Nevertheless, handling and tagging protocols were designed and supervised by Dr. L Dagorn, who holds a diploma on animal experimentation and surgery granted by the Ecole Nationale Vétérinaire de Nantes, France. The commercial fishing vessel supporting this research holds an appropriate fishing license provided by the Ministry of Fisheries and Aquaculture to operate on Brazilian and adjacent waters (SFPA-RN—NUP: 21040.000495/99-11).

### Tagging

A total of 11 oceanic whitetip sharks were tagged with pop-up satellite archival tags (PAT), manufactured by Wildlife Computers (Redmond, USA), in the South Atlantic Ocean. From these, 7 reported data to the satellites as scheduled, 1 reported prematurely and 3 never reported (Table 1). Two models of PAT were used: 7 MK10s and 4 miniPATs, with all of the non-reporting tags being Mk10s. Both models were set to collect data on depth, water temperature and ambient light level (for estimation of geolocation) every 10 seconds. The Mk10, however, only transmitted a summary of that data in the form of depth and temperature histograms, while the MiniPATs transmitted time series data at a resolution of 5 minutes.

**Table 1:** Meta data of the oceanic whitetip sharks tagged in the western Atlantic Ocean.

					TAGGING			POP-UP			
ID	TL (cm)	SEX	TAG	PROGRAMMED	DATE	LAT	LON	DATE	LAT	LON	DURATION
AOCS 1	135	F	MK10	60 days	29/01/2010	-0.995	-30.88	30/03/2010	-1.914	-34.637	60
AOCS 2	152	M	MK10	90 days	05/02/2010	0.158	-29.777	06/05/2010	-0.218	-38.255	90
AOCS 3	167	M	MK10**	180 days	16/01/2011	-0.139	-34.218	10/07/2011	-3.802	-32.466	178
AOCS 4	197*	F	miniPAT	140 days	06/12/2011	-3.589	-34.918	25/04/2012	-18.754	-35.771	141
AOCS 5	180*	F	miniPAT	140 days	01/03/2012	-0.501	-37.354	20/07/2012	3.215	-41.015	141
AOCS 6	134	F	miniPAT	100 days	02/03/2012	-0.736	-37.534	11/06/2012	-0.598	-36.235	101
AOCS 7	161	F	miniPAT	100 days	02/03/2012	-0.435	-37.629	14/06/2012	1.306	-35.345	104
AOCS 8	100	F	MK10	90 days	05/03/2012	-2.403	-37.983	21/05/2012	4.492	-32.624	77
-	140	F	MK10	180 days	20/01/2011	-1.3889	-34.533				
-	168	M	MK10	90 days	06/03/2012	1.763	-42.977	**NEVER REPORTED**			
-	100	M	MK10	180 days	13/03/2012	-0.0565	-38.140				

*Individuals larger than the size at first maturity (180 cm).

**Recovered tag.

The first two Mk10 tags (AOCS1 and AOCS2) were programmed to summarize the collected data into one-hour histograms while all others were set to generate six-hour histograms. One MK10 was recovered (AOCS3) allowing the complete time series, with 10-second resolution, to be accessed. The miniPATs were programmed to transmit only depth time series data and not temperature. Temperature data were received as a daily histogram (24 hours). Both tag models transmitted light data in the form of two daily light curves (representing dawn and dusk) that were used to reconstruct geographic positions. The MiniPATs and the recovered MK10 also provided a daily analysis of the surface mixed layer depth and the amount of time the tagged shark spent therein.

All OCS were caught off the Northeast coast of Brazil by a commercial tuna longliner and tagging was conducted by an on-board observer with the help of the crew. Sharks were brought onboard to be measured and tagged and were out of the water for no longer than five minutes prior to release. Tags were attached at the base of the first dorsal fin using a loop of polyamide monofilament (2.0 mm) passed through a silicon tube, to minimize friction related injuries. Tagging locations were recorded using the vessel’s global positioning system (GPS).

### Vertical distribution

Depth data were analyzed in relation to the different periods of the day to account for diel movement patterns. Day, night, dawn and dusk data were grouped according to local times, which were estimated using the calculation procedure available from NOAA (http://www.srrb.noaa.gov/highlights/sunrise/sunrise.html). Data from the Mk10 tag that were summarized into six-hour histograms could not be grouped. Day was defined as the period between sunrise and sunset, and night as the period between astronomical dusk and astronomical dawn. Dusk comprised the hours between sunset and astronomical dusk, while dawn comprised the hours between astronomical dawn and sunrise. To compare daytime and nighttime depth distributions, Pearson’s chi-squared test was performed at 95% confidence level. Crepuscular periods (dawn and dusk) were omitted from this analysis.

### Horizontal Movements

The horizontal movements of tagged sharks were estimated by processing the data received from the Argos satellite system using the manufacturer light-based geolocation software (WC-GPE: Global Position Estimator Program suite, available at: www.wildlifecomputers.com). Longitude is estimated from the time of local noon and latitude from the length of the day through the dawn and dusk symmetry method. In order to minimize the errors usually associated to this geolocation estimation [12,13], the tracks were post-processed using the IKNOS Walk model [14]. In this approach, tracks are corrected and interpolated at fixed intervals by bootstrapping random walk particles. The locations are estimated from a cloud of weighted particles and corrections can be applied based on known constraints or available data.

In the present study, the tracks were interpolated to obtain one position a day and were prevented from crossing land. A maximum speed threshold was also implemented into the model. Starting from 3 km/h, several speeds were tested up to 10 km/h (by 1 km/h increments). The maximum speed of 9 km/h was selected based on the end point criteria, meaning this maximum speed was the slowest speed that allowed all tracks to end at the known pop-up point. The error around estimated positions is displayed by 50 alternative positions for each point, representing minimum and maximum latitudes and longitudes.

To estimate the areas of high utilization, a Two-Dimensional Kernel Density Estimation was applied to the cloud of alternative points (error). Data from all individuals were combined in this analysis, conducted using the MASS package [15] in the R environment [16]. The bandwidth was chosen via Normal Reference Distribution. In order to expose areas of high utilization, density values smaller than 0.01 were excluded from the plot.

### Fisheries data

Fishing effort data from the Brazilian tuna longline fleet between 1999 and 2011, totaling 65,277 sets, were used to assess the vulnerability of oceanic whitetip sharks to this fishery. The Brazilian fleet operates in a wide area of the equatorial and southwestern Atlantic Ocean and uses two distinct fishing strategies commonly referred to as “Japanese” (JAP) and “Spanish” (SPA). In general, vessels fishing with the Spanish strategy target swordfish (*Xiphias gladius*) and set surface longlines (down to 100 m) at night, using lightsticks and squid as bait. Alternatively, vessels fishing with the Japanese strategy target various tuna species and set deep longlines (down to more than 200 m) early in the morning, using small pelagic fish as bait (mainly mackerel) [17]. Fishing effort was represented by the number of hooks deployed, grouped into 5° squares for spatial analyses.

Nominal catch per unit of effort (CPUE) data were analyzed to account for possible seasonal variations. A subsample of the dataset previously analyzed by Tolotti et al. [17] was considered for this purpose. This dataset was collected by the Brazilian Observer Program from foreign-chartered tuna longline vessels between 2004 and 2010. Spatial bounds for this data were selected based on the shark high utilization areas (from tag data), as well as on the area where the fishing effort was concentrated. The CPUE represented the number of OCS/1,000 hooks per quarter. Mean values were calculated using the sum of all catch and all effort in each quarter. Data were also grouped according to the fishing strategy. Two-sample Kolmogorov-Smirnov tests were used for inter-quarter comparisons at a 95% confidence level.

To evaluate the spatial overlap between fisheries and tagging data precisely, it would be necessary to use a CPUE index that accounts for the depth distribution of the hooks. In the absence of such data, an occurrence index was adopted. For this analysis, data were also grouped into 5° squares and by quarter. For each square, the number of sets with the presence of OCS were summed and then divided by the total number of observed sets. The occurrence values were then paired with the Kernel densities (also grouped by quarters and 5° × 5° squares). For each square, density values were summed. A Spearman's Rank test was conducted to verify possible correlation between fisheries and tagging data (occurrence and Kernel density). The non-parametric Spearman's Rank was chosen after verifying that the two datasets did not conform to a bivariate normal distribution (Royston's Multivariate Normality Test, p = 9.217239e-06).

## Results

### Tag performance

In total, 719 geolocation days, 1,643,249 depth records and 1,537,920 temperature readings from the 8 reporting tags were analyzed. Time at depth histograms totaled 951 and time at temperature 1,340. Overall, the miniPATs had higher reception rates than the MK10s for both general and geolocation data (Table 2). Except for one miniPAT (OCS5), reception rates were approximately 80% for general data and over 97% for geolocation. The OCS5 miniPAT stopped collecting data after 104 days of deployment, but popped to the surface and transmitted the data after 140 days as scheduled. Nonetheless, these data sets were almost entirely received (86.2% and 99.0% of general and geolocation data, respectively). The MK10 tags varied considerably in their reporting rates, and ranged from as low as 7% to up to 53% of the general data and from 10% to 63% for geolocation data (Table 2).

**Table 2:** Data reception of pop-up satellite archival tags and distances traveled by oceanic whitetip sharks tagged in the western Atlantic Ocean between 2010 and 2012.

ID	Tag	General data (%)	Geolocation (%)	Total track length (km)	Straight line distance (km)	Movement rate (km.day^-1^)
AOCS 1	MK10	07.63	10.00	1701	439	28
AOCS 2	MK10	31.39	63.33	6217	949	69
AOCS 3*	MK10	100.00	100.00	19043	493	107
AOCS 4	miniPAT	79.51	98.58	14273	1733	101
AOCS 5**	miniPAT	63.56	73.05	11355	580	81
AOCS 6	miniPAT	82.57	98.02	12117	145	120
AOCS 7	miniPAT	80.56	97.12	11250	305	108
AOCS 8	MK10	53.57	46.75	7118	966	92

*Recovered.

**Tag stopped recording data after 104 days of deployment and reception rates for the period were 86.17 and 99.04%.

### Temperature preferences and vertical distribution

**Fig 1:**
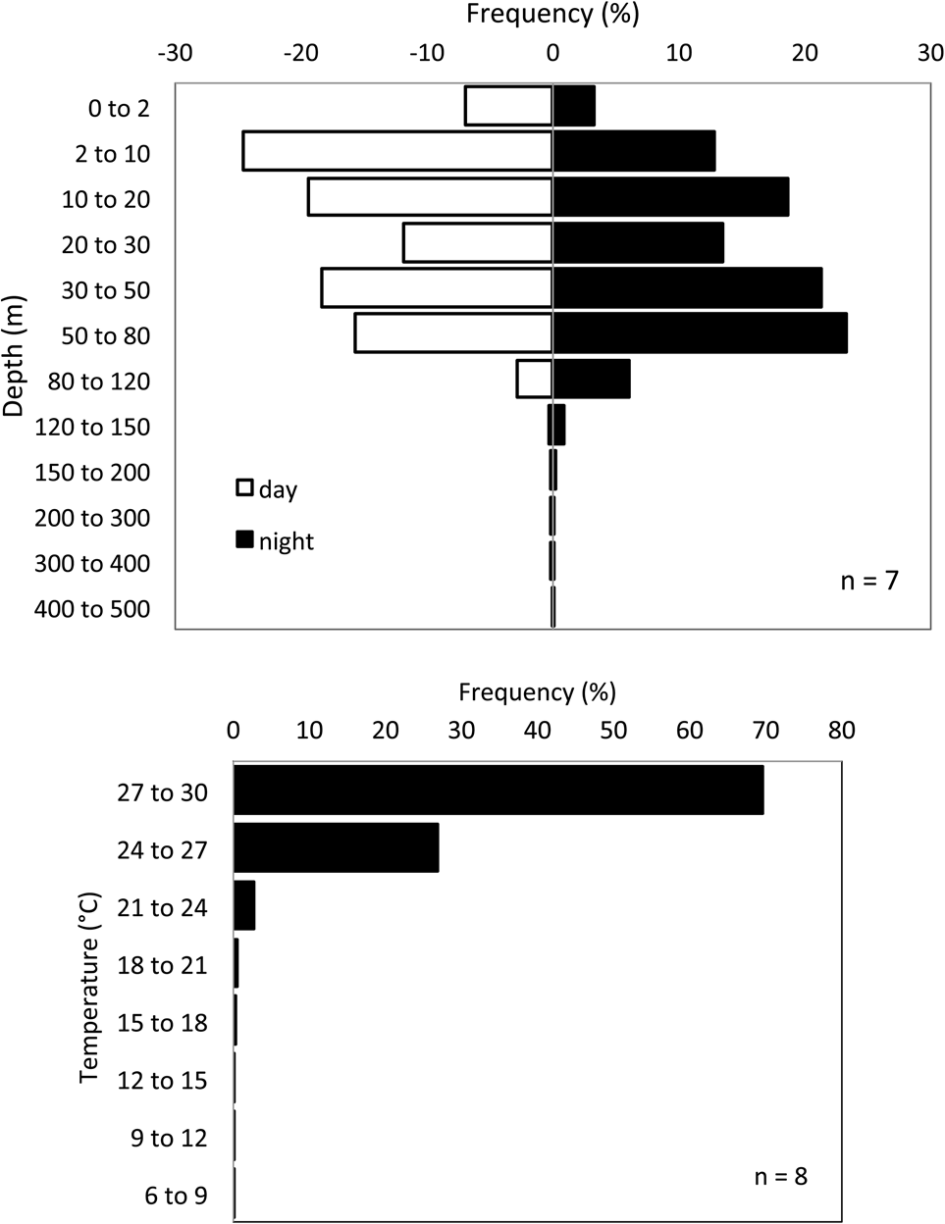
Depth and temperature preferences of oceanic whitetip sharks tagged in the western Atlantic Ocean between 2010 and 2012. Depth frequencies includes sharks OCS1 to 7 and temperature frequency includes data on all sharks.

Oceanic whitetip sharks exhibited a strong preference for warm and shallow waters. Tagged sharks remained at temperatures between 24 and 30°C for approximately 96% of the monitoring period (Fig 1). The time spent inside the mixed layer was very similar for all sharks, regardless of their size, and it varied between 70 and 83% (Table 3). AOCS4 and 7 entered the coldest waters of all tagged sharks. The minimum recorded temperatures were 8.2°C, with a corresponding depth of 368 m, for AOCS4, and 8.8°C at 448 m, for AOCS7. The latter also represented the deepest recorded dive of this study.

**Table 3:** Average time spent within the mixed surface layer by oceanic whitetip sharks tagged in the western Atlantic Ocean.

%MixL	AOCS 3	AOCS 4	AOCS 5	AOCS 6	AOCS 7
mean	76.97	70.43	73.87	73.47	83.56
SD	18.11	13.45	16.52	18.04	10.86
SE	0.68	1.71	1.71	1.87	1.14
1^st^ quart.	67.00	61.00	64.00	61.00	79.50
median	80.00	72.00	79.00	77.00	85.00
3^rd^ quart.	92.00	79.00	86.00	88.00	91.50

For more than 95% of the monitoring period tagged sharks remained above 120 m, during both day and night periods (Fig 1). The aggregated data showed similar frequency distributions between day and night, although during the day shallower depth intervals were slightly more frequent, whilst the night distribution showed higher frequencies in deeper intervals. These differences, however, were not statistically significant (χ^2^ test, p = 0.6727).

### Horizontal movements

**Fig 2:**
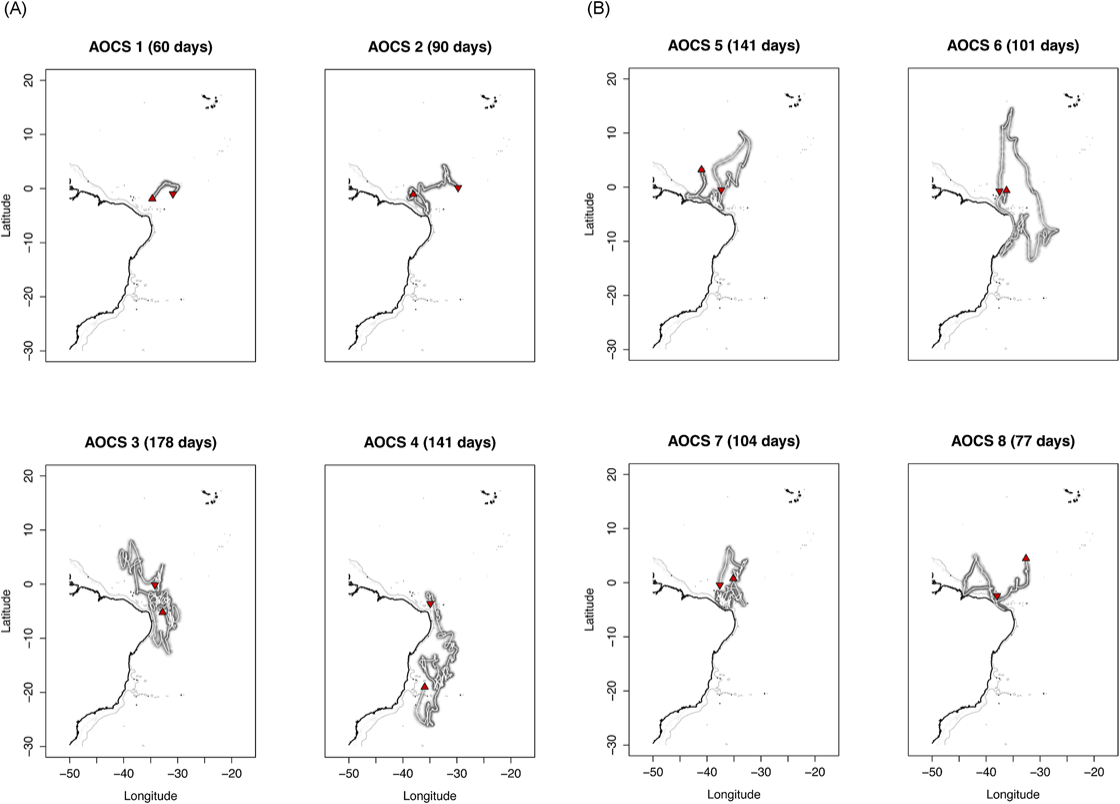
Post-processed tracks of oceanic whitetip sharks tagged in the western Atlantic Ocean. The downward triangles represent the tagging position and the upward triangles the end of the track. The grey-shaded area represents the error around estimated positions. (A) Oceanic whitetip sharks tagged in 2010 and 2011.(B) Oceanic whitetip sharks tagged in March

**Fig 3:**
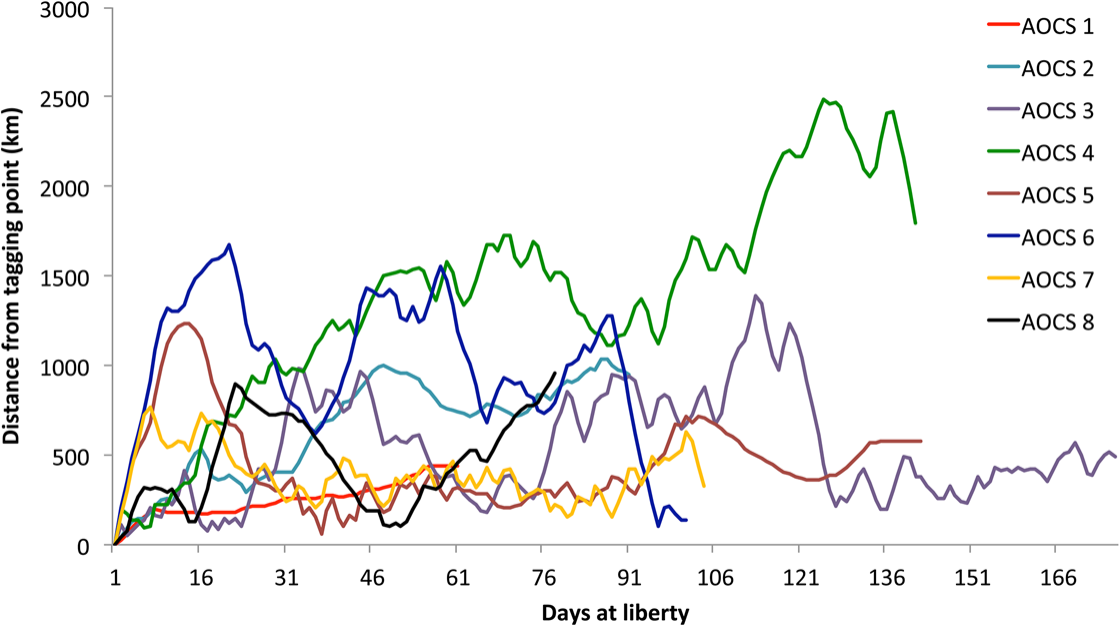
Distance from the tagging position as a function of days at liberty for oceanic whitetip sharks tagged in the western Atlantic Ocean between 2010 and 2012.

All sharks were tagged close to the equator and although individuals tended to travel long distances the pop-up positions were relatively close to the tagging locations (Fig 2A and 2B). The individual AOCS6, for example, moved as far as 12,117 km during the tag deployment, but the distance between tagging and pop-up locations was only 145 km (Table 2). A similar pattern was observed on AOCS1, 3, 5 and 7. AOCS5 and 7 stayed within 500 km of the tagging position during most of their track, while AOCS1 remained in this range for the entirety of its monitoring period (Fig 3). AOCS3 made more extensive movements but returned to de 500 km range after 127 days at liberty (Fig 3). Only one shark made extended movements to the south (AOCS4), reaching latitudes below 10°S. This shark moved south shortly after tagging and did not return to the tagging area within the 141 days of monitoring. The last part of its track, however, shows a northward movement (Fig 2A).

Total track lengths varied from 1,701 km, represented by the shortest deployment period (60 days), to 19,043 km, represented by the longest (178 days). Daily displacements varied considerably among sharks and ranged from 28 to 120 Km.day^-1^ (Table 2). Overall, the horizontal movements were more pronounced in terms of latitude, whereas longitudinal movements were more restricted, not surpassing the 25° meridian.

### Fishing effort and CPUE

**Fig 4:**
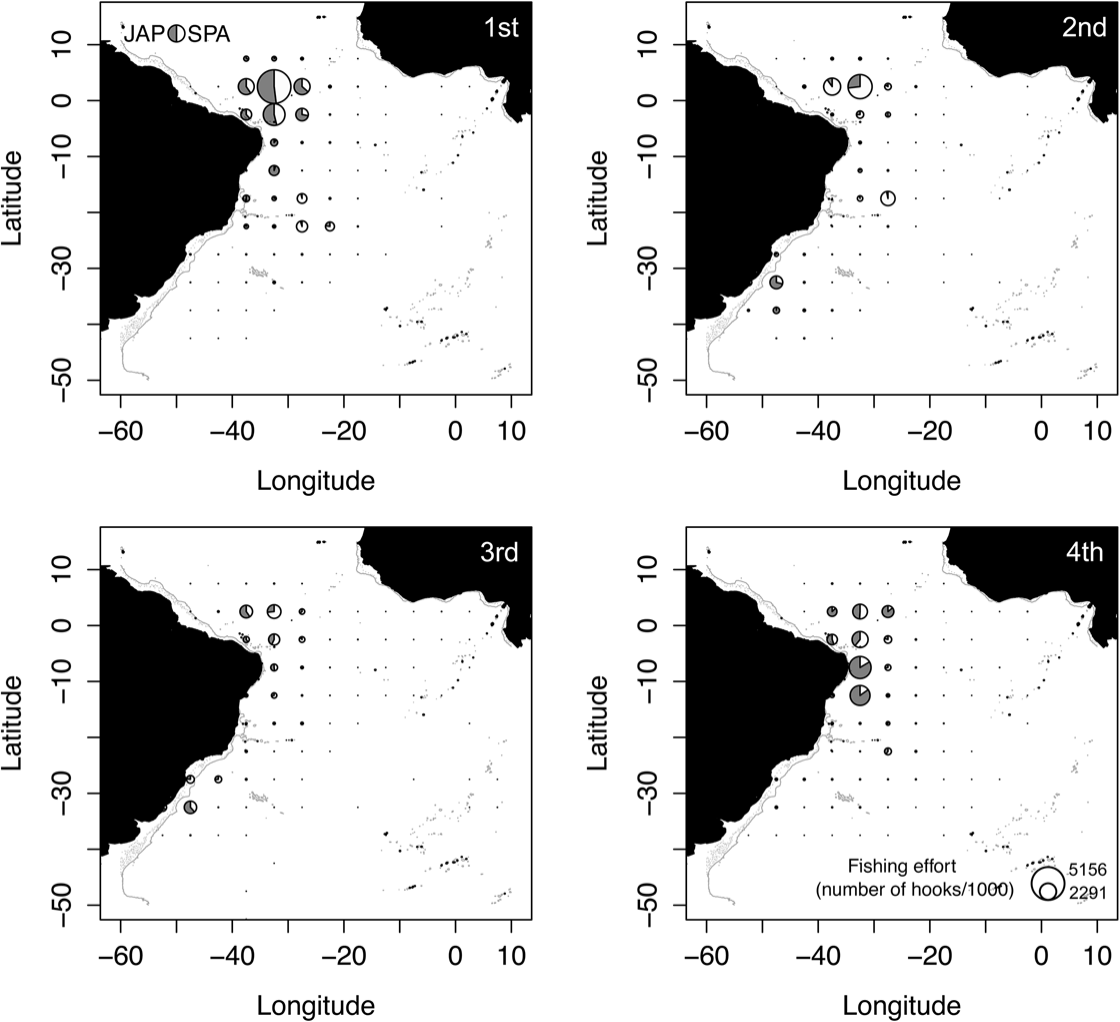
Fishing effort of the Brazilian longline fleet represented by the number of hooks deployed from 1999 to 2011.

**Fig 5:**
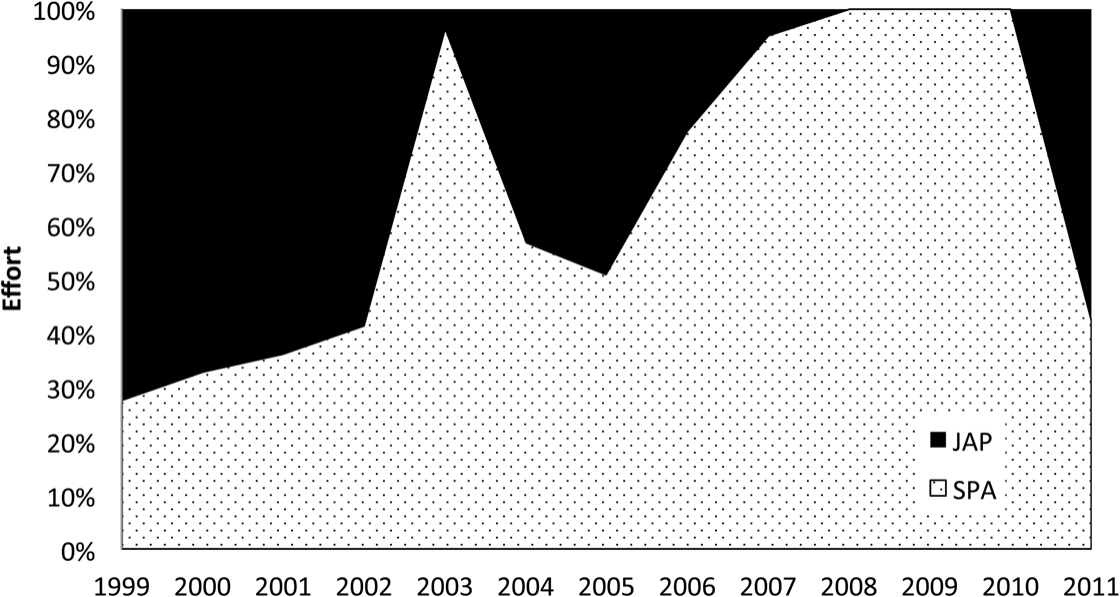
Proportion of the two different fishing strategies of the Brazilian longline fleet between 1999 to 2011.

Between 1999 and 2011 the Brazilian tuna longline fleet deployed a total of 102,201,242 hooks. The sets were widely distributed in the equatorial and southwestern Atlantic Ocean, ranging from 10°N to 65°S in latitude and from 007°E to 055°W in longitude. The area with the highest effort concentration was bound by the 5°N and the 15°S parallels and by the 040°W and 035°W meridians (Fig 4). Despite the wide distribution of fishing sets, this area of highly concentrated effort is clearly evident. For both fishing strategies, SPA and JAP, most hooks were deployed within this area of concentrated effort. In contrast, the proportion of hooks deployed by fishing strategy varied considerably over time (Fig 5). From 1999 to 2002 the Japanese strategy accounted for the majority of hooks deployed, starting at 70% and gradually decreasing to 60%. From 2003 onwards, the Spanish strategy was consistently dominant, although some fluctuations were observed. During the years of 2008, 2009 and 2010 the Brazilian fleet consisted entirely of vessels fishing with Spanish strategy.

**Fig 6:**
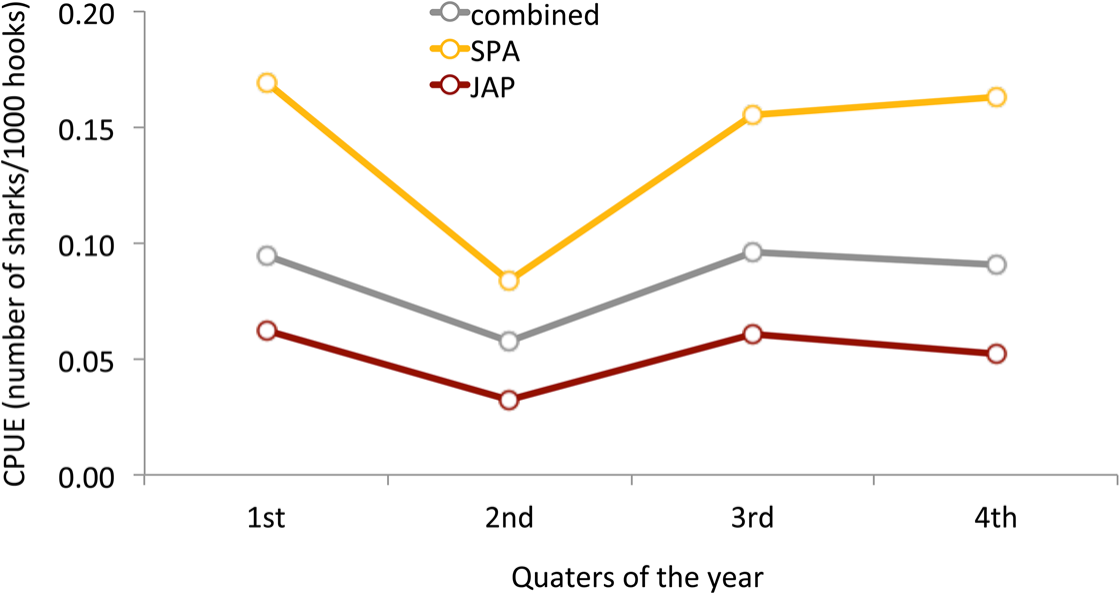
Mean CPUE values of oceanic whitetip sharks caught by foreign tuna longline vessels chartered by Brazil operating from 2004 to 2010.

Mean CPUE values were similar throughout the year with slight differences in the second quarter. Overall CPUE values ranged between 0.08 and 0.17 sharks/1000 hooks for SPA and from 0.03 to 0.06 for JAP (Fig 6). The same trend was observed in both fishing strategies, although Spanish CPUE values were always considerably higher. For this fishing strategy the mean CPUE value of the second quarter was significantly lower compared to the other quarters (Table 4).

**Table 4:** P-values of Two-sample Kolmogorov-Smirnov tests comparing CPUE values for all quarters of the year.

Quaters	*p* (combined)	*p* (SPA)	*p* (JAP)
1^st^ x 2^nd^	0.0926	0.0195*	0.6940
1^st^ x 3^rd^	0.5758	0.9505	0.9572
1^st^ x 4^th^	0.9835	0.9921	0.8832
2^nd^ x 3^rd^	0.0839	0.0584	0.5765
2^nd^ x 4^th^	0.2976	0.0250*	0.9849
3^rd^ x 4^th^	0.9957	1.0000	0.8950

*Statistically different, p = 0.05.

### Spatial dynamics of occurrence and Kernel density data

**Fig 7:**
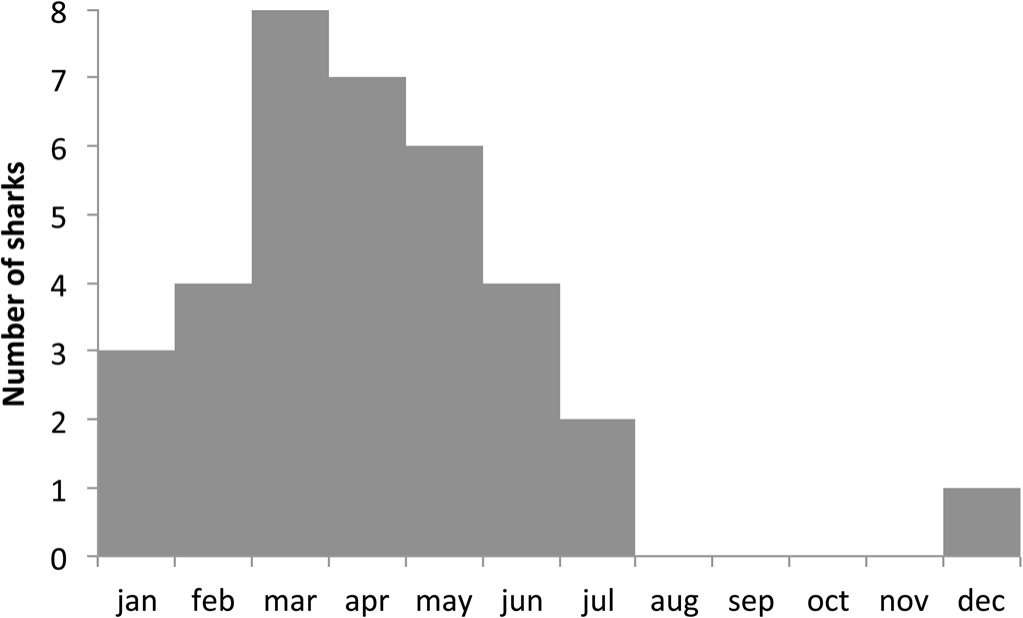
Number of oceanic whitetip sharks monitored per month.

**Fig 8:**
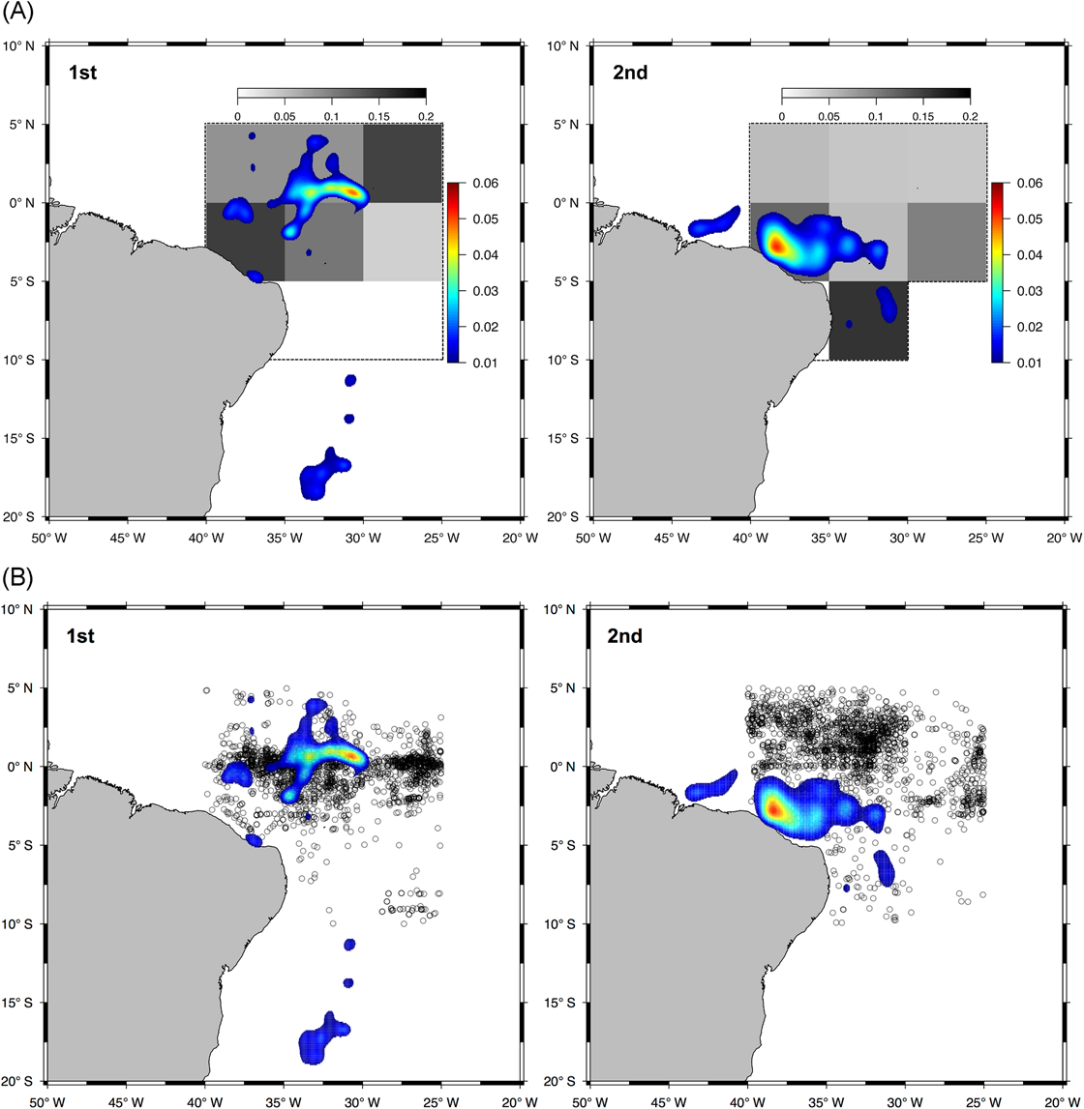
Kernel density estimation of post-processed tracks showing the areas of high utilization by oceanic whitetip sharks tagged in the western Atlantic Ocean between 2010 and 2012. The left panel represents the 1^st^ quarter of the year and the right represents the 2^nd^. (A) Values of occurrence per set (grey scale) for the species from foreign tuna longline vessels chartered by Brazil operating from 2004 to 2010. Doted lines mark the area where the occurrence data were analyzed. (B) Fishing sets locations (circles) from foreign tuna longline vessels chartered by Brazil operating from 2004 to 2010

Kernel densities were only estimated for the first two quarters of the year due to the concentration of tagging data during this period (Fig 7). The Kernel densities of combined tracks revealed two distinct areas of high utilization, one during each quarter of the year (Fig 8A). In the first quarter, high densities were concentrated very close to the Equator between the 035°W and the 030°W parallels. During the second quarter the highest density values were located closer to the coast just off northeast Brazil above the *Cabo Calcanhar*. Both high-utilization areas fall inside the high fishing effort zone, although second quarter sets were concentrated north of the OCS utilization hot spot (Fig 8B).

The spatial distribution of OCS occurrence differed between the first two quarters of the year. Overall, high occurrence values appeared in the area towards the north of the study zone in the 1^st^ quarter, while in the 2^nd^ quarter high values were located towards the south (Fig 8A). The square located just off *Cabo Calcanhar* was the only area that showed similar values during both quarters. The greatest difference was seen in the southernmost square, with zero occurrences in the 1^st^ quarter and 0.16 OCS presences per set in the 2^nd^.

A strong spatial correlation between occurrence and Kernel density estimation is not clearly evident in Fig 8A, although it is notable that high-density hotspots do fall inside squares with higher occurrence. Likewise, squares with very low occurrence (<0.05) have none or very little utilization according with the Kernel density estimation. This apparent positive correlation was confirmed by the Spearman's rank test, which rejected the null hypothesis that kernel density values and CPUE were not correlated (Spearman's Rank Test p = 0.02009, rho = 0.5919304).

## Discussion

### Vertical vulnerability

The vertical movements observed in the present study are in agreement with the existing literature, which describes the oceanic whitetip shark as an epipelagic predator of warm tropical waters [2] and places the species in the group of pelagic fishes that spend the majority of their time in the upper uniform-temperature surface layer, rarely descending to waters below 20°C [18]. Through the use of electronic tags, two recent studies have also provided evidence to confirm these general statements. Musyl et al. [19], working near Hawaii, found that OCS were largely confined to the mixed layer (~120 m), spending > 95% of their time at temperatures within 2°C of the sea surface temperature (SST). OCS from the North Atlantic (Bahamas) exhibited a similar vertical pattern, essentially occupying the upper 125 m of the epipelagic zone at temperatures close to the SST [20].

The oceanic whitetip shark’s strong preference for shallow and warm waters is also reflected on the depths of the longline hooks where the species is most commonly caught. Nakano et al. [21], for instance, concluded that the catch rates of OCS increased significantly with the decrease of hook depth. In a more specific study, data from the Brazilian longline fleet showed that the CPUE for OCS tended to be lower for the vessels targeting tuna, which operate with deeper longlines (down to 200 m), than those targeting swordfish, which operate with shallower gear (> 100 m) [17]. A sample of this data set, shown in Fig 7, further highlights the greater impact of the shallow Spanish fishing strategy on OCS CPUE values. This result comes with little surprise given that the depth range of the gear used in this fishing strategy corresponds exactly to the species vertical distribution.

This vertical overlap is particularly concerning when the majority of the Brazilian fleet operates with shallow longlines exactly in the area where the species can be most readily found. Although the proportion between the two fishing strategies (JAP-deep and SPA-shallow) in the Brazilian fleet varies considerably over the years, these fluctuations are mainly due to temporary fishing agreements between the Brazilian government and foreign companies [22]. The permanent Brazilian fleet, however, typically operates with shallow gear, primarily around the equator and close to seamounts [23], which means the vertical preferred habitat of the oceanic whitetip shark is extensively exploited.

The results from the present study, combined with other recently published works, show that the vertical distribution of the oceanic whitetip shark is well defined. Despite the clear need for further data to fill the extensive knowledge gaps on the ecology of the species, this information is already of great importance for defining mitigation measures. A ban on retaining, landing, storing or selling this species has been established by all tuna RFMOs. This measure, however, does not preclude fishers from catching oceanic whitetip sharks. Practical mitigation methods, such as eliminating shallow hooks (above 100 m) on longlines, require particular attention. The removal of shallower hooks has already shown promising results in reducing catch rates of several epipelagic bycatch species [24]. It is important to bear in mind, however, that mitigation measures can generate impacts on other species and so both their positive and negative impacts must be carefully evaluated [25].

### Horizontal vulnerability

Oceanic whitetip sharks tagged in the southwestern Atlantic Ocean appear to have a certain degree of site fidelity to the area off northeast Brazil around the equator, where all of their tracks started. Five out of eight sharks have ended their tracks relatively close to their starting points, even after traveling several thousand kilometers. Three of these sharks also remained within 500 km of the tagging locations during most of the tracking period. Although time limitations prevent definitive conclusions from being drawn, a recent study conducted in the Bahamas (northwestern Atlantic) also reported that OCS returned to the tagging area after long migrations [20]. The Bahamian study gathered data from 10 OCS with deployment periods up to 245 days. In the year following their study, scuba divers also spotted one of the tagged sharks near the tagging location [20]. The authors considered this returning behavior to fall under the definition of philopatry, which is the tendency of an organism to return or remain near a particular site [26]. The findings presented here further support this theory.

Another interesting observation is that oceanic whitetips from both studies (Northwestern and Southwestern Atlantic) did not mix. More data is certainly required before a conclusion can be reached, but this result could be an indication that northern and southern populations might well be separated. The recently developed “electronic spaghetti tag” could be a useful and cost-effective tool to investigate this matter. This new satellite tag does not record any data, but simply reports where it pops off, providing fisheries-independent measures of dispersal patterns and migration [27]. Fine scale genetic studies using microsatellite loci could also shed light on this matter [28]. If these hypotheses hold, well-defined populations and philopatric behavior can have substantial implications for the conservation of this species and suggest that the oceanic whitetip shark could benefit, not only from global management measures, but from local measures as well.

The distribution of the effort from the Brazilian longline fleet raises important concerns regarding the species’ vulnerability to fisheries, especially given its depleted population status [29–31]. The Kernel Density estimates indicate that the high utilization areas fall exactly within the area where the fishing pressure is considerably higher. The region off northeast Brazil, where OCS seems to have some degree of philopatry, is rich in oceanic features such as islands, banks and seamounts, mostly belonging to the North Brazil and Fernando de Noronha Chains, located between 2° and 4° South and 32° and 40° West [32]. The areas surrounding these features are considered important fishing grounds for tuna and tuna-like species, including sharks [23,33].

Interestingly, when looking at the study area at a finer scale the kernel hot spot for the second quarter appears to be slightly outside of the highest fishing pressure area (Fig 8B). In this quarter, the high-utilization hot spot of oceanic whitetip sharks is located just off northeast Brazil above the *Cabo Calcanhar* and below the equator, while most of the fishing sets at this time of the year were made just above the equator. This lack of overlap between the species high-utilization area and fisheries highest effort could explain the significantly lower CPUE during the second quarter as seen in Fig 6. A much stronger overlap is seen on the first quarter, which had a higher average CPUE. Considering the time limitation of the study (tagging data concentrated on the first semester) and the few individuals tagged, it is interesting to find a spatial correlation between fisheries and tagging data. The philopatric behavior combined with the fact that tagging was conducted through commercial fishing vessels, might have contributed for this result. Tagging oceanic whitetip sharks outside of the major fishing zone would provide essential information for validating these findings. In any case, the correlation found here highlights the potential that tagging experiments hold for providing knowledge for the design of spatio-temporal management measures.

More data are clearly needed to understand the spatial dynamics of oceanic whitetip sharks in the southwestern and equatorial Atlantic and more tagging experiments should be encouraged. Future studies should focus on covering the entire year cycle through both long deployment periods and tagging during different seasons. Improved fisheries monitoring is also of extreme importance and observer programs therefore require expansion. This study also highlights how fisheries dependent and independent data sources can complement one another.
